# A Straightforward Approach to Analyze Skeletal Muscle MRI in Limb-Girdle Muscular Dystrophy for Differential Diagnosis: A Systematic Review

**DOI:** 10.3390/muscles2040029

**Published:** 2023-11-08

**Authors:** Ryo Morishima, Benedikt Schoser

**Affiliations:** 1 Department of Neurology, Tokyo Metropolitan Neurological Hospital, Musashidai 2-6-1, Fuchu 183-0042, Tokyo, Japan; 2 Friedrich-Baur-Institute, Department of Neurology LMU Clinic Munich, Ziemssenstr. 1, 80336 Munich, Bavaria, Germany; benedikt.schoser@med.uni-muenchen.de

**Keywords:** limb-girdle muscular dystrophy, skeletal muscle MRI, muscle dystrophies, systematic review

## Abstract

Skeletal muscle MRI studies in limb-girdle muscular dystrophy (LGMD) have increased over the past decades, improving the utility of MRI as a differential diagnostic tool. Nevertheless, the relative rarity of individual genotypes limits the scope of what each study can address, making it challenging to obtain a comprehensive overview of the MRI image of this splintered group. Furthermore, MRI studies have varied in their methods for assessing fat infiltration, which is essential in skeletal muscle MRI evaluation. It stayed problematic and impeded attempts to integrate multiple studies to cover the core MRI features of a distinct LGMD. In this study, we conducted a systematic review of LGMD in adults published until April 2023; 935 references were screened in PubMed and EMBASE, searches of the gray literature, and additional records were added during the screening process. Finally, 39 studies were included in our final analysis. We attempted to quantitatively synthesize the MRI data sets from the 39 individual studies. Finally, we illustrated ideal and simple MRI muscle involvement patterns of six representative LGMD genotypes. Our summary synthesis reveals a distinct distribution pattern of affected muscles by LGMD genotypes, which may be helpful for a quick first-tier differential diagnosis guiding genetic diagnostics.

## 1. Introduction

Limb-girdle muscular dystrophies (LGMDs) belong to hereditary myopathies, a group of diseases clinically characterized by progressive atrophy of the proximal muscles and varying degrees of atrophy in other parts of the body’s skeletal muscle. More than 30 genotypes are known, ranging from relatively common forms to ultrarare entities [1].

The diagnosis of hereditary myopathy is based on clinical diagnosis, muscle pathology, and the final confirmation by gene sequencing analysis. This diagnostic chain has remained chiefly unchanged till today. However, genetic diagnosis and muscle imaging have been added to this classic diagnostic work-up and are now the preferred first-tier diagnostic decision-makers. Skeletal muscle MRI has become a powerful tool for differential diagnosis because of advances in clinical knowledge and research [2]. Skeletal muscle MRI allows easy identification of individual skeletal muscles, guiding a straightforward determination of distribution pattern recognition of affected muscles. The most frequently used parameter for skeletal muscle MRI is the degree of fat infiltration on t1-weighted images, followed by the evaluation of myoedema on fat-suppressed images such as STIR. In recent years, studies have been conducted to quantitatively analyze the degree of fatty infiltration from MRI images.

Nevertheless, subtypes of LGMDs have yet to reach the stage where each form can be differentiated from the others, partly because of the numerous subtypes, the different genotypes, and the different timing of progress of the natural course of these diseases contained therein. Indeed, although the number of studies addressing this issue has increased over the past decade [3,4], the results of each study could not be easily compared because each study used a different evaluation method or only described the subjectivity of the author’s evaluation. Two systematic reviews have been conducted on this topic, one reviewing myopathy in general when there were limited studies on MRI images of LGMD, and the other including children and many single case reports, limiting the quantitative evaluation and the likelihood analysis of the individual muscles. Our systematic review focuses exclusively on LGMDs in adults, emphasizing comparing studies in an easy-to-understand manner for practical use in clinical practice.

This study aims to integrate previous studies on the major forms of LGMD to clarify muscle selectivity patterns and facilitate differential diagnosis when LGMD is suspected in the adult setting.

## 2. Results

### 2.1. Study Selection

**Figure 1:**
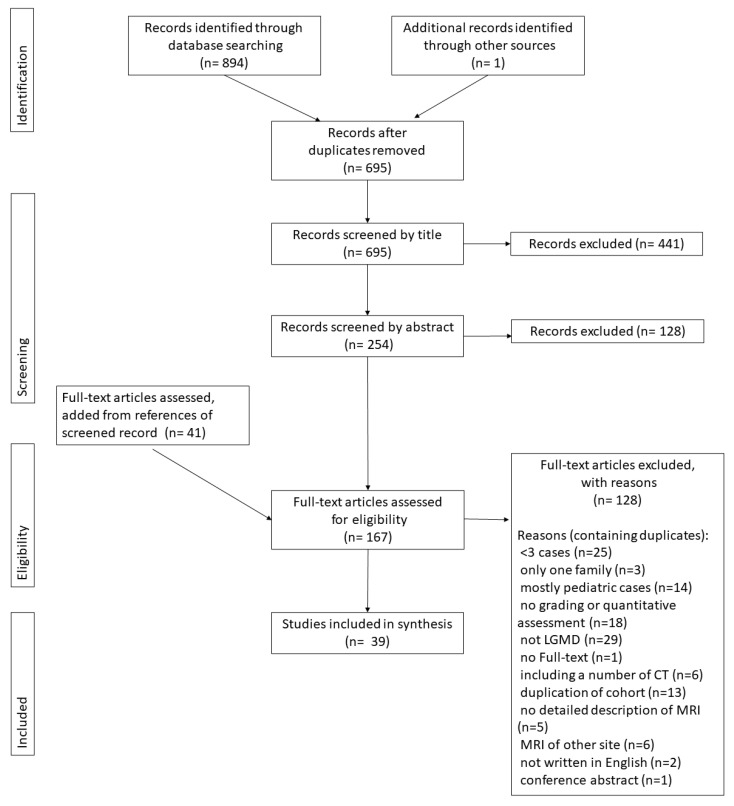
PRISMA flowchart of the selection of the studies for this review.

The studies were selected to be included as follows (Figure 1). The initial search yielded 355 hits in MEDLINE and 529 hits in EMBASE. Of these 894 hits, 199 duplicates were excluded, leaving 695 for initial screening. The database search was conducted on 20 April 2023. A search of Cochrane did not find any relevant studies, and a search of the grey literature found only one study in Open-Grey and zero in the Grey Literature Report, but no new studies to add.

From this point, screening by title proceeded based on the exclusion criteria, and 441 studies were excluded. The remaining 254 studies were screened using abstracts, and 128 were excluded. The full-text screening was conducted for the remaining 126 studies. The reference list of all studies was checked during the full-text screening process, and 41 additional studies were added that had not been caught in previous searches, bringing the total number of studies that underwent full-text screening to 167. After the full-text screening, 128 studies were excluded. In cases with multiple studies dealing with the same cohort, the study with the most objective description using semi-quantitative or quantitative evaluation methods was included.

Finally, 39 studies were used for analysis and synthesis. Of these, 32 used the grading system, 11 were qualitative studies, and four mentioned both evaluation methods.

The following is a breakdown of the 32 analyzed, which have been evaluated by the grading system; LGMD R1 calpain3-related (R1(CAPN)) 9 [5,6,7,8,9,10,11,12,13], R2(DYSF) 11 [5,11,12,13,14,15,16,17,18,19,20], R3–6(SGC) 4 [11,12,21,22], LGMD R7 telethonin-related (R7(TCAP)) 1 [23], LGMD R9 FKRP-related (R9(FKRP)) 4 [11,12,24,25], R12(ANO5) 5 [26,27,28,29,30], LGMD R14 POMT2-related (R14(POMT2)) 1 [31], LGMD R19 GMPPB-related (R19(GMPPB)) 3 [32,33,34], LGMD D3 HNRNPDL-related (D3(HNRNPDL)) 1 [35], LGMD D5 collagen 6-related and LGMD R22 collagen 6-related (D5/R12(COL6)) 1 [36].

Of these, 24 presented the data in calculable form rather than only in textual representation, as follows: R1 (CAPN) 7 [5,6,7,8,10,11,13], R2 (DYSF) 9 [5,11,13,14,15,16,17,19,20], R3–6 (SGC) 3 [11,21,22], R7 (TCAP) 1 [23], R9 (FKRP) 2 [11,24], R12 (ANO5) 5 [26,27,28,29,30]. R19 (GMPPB) 2 [32,34].

In addition, the studies in which quantitative assessments have been conducted are as follows: R1 (CAPN) 3 [6,37,38], R2 (DYSF) 3 [16,37,39], R9 (FKRP) 4 [24,39,40,41], R12 (ANO5) 2 [26,42], D5/R12 (COL6) 1 [43].

Of the above, quantitative synthesis was performed for the following genotypes, which included multiple studies: R1 (CAPN), R2 (DYSF), R3–6 (SGC), R9 (FKRP), R12 (ANO5), R19 (GMPPB).

### 2.2. Demographic Data

The regions where MRIs were performed, mean age, mean disease duration, and gender are shown in (Supplementary Table S1). The data for the mean values of age, sex, and disease duration are combined in (Table 1). The regions studied were predominantly within Europe, followed by a small number of Asian countries, but the Americas, Oceania, and Japan were not included at all.

Mean age was stated or calculable from the data provided in all but one study in the quantitative studies [39]. Standard deviations were challenging to perform synthetic comparisons on because of the large quantity of missing data, especially in the most numerous R2 (DYSF). Sex was nearly equal for men and women in many diseases with a sufficient sample size, but in R12 (ANO5), 70% of the patients were male.

Although it was difficult to synthesize the overall disease duration due to missing data, the four disease types had a mean duration of 12–14 years, except R9 (FKRP) and R19 (GMPPB), which had a mean duration of 20 years.

### 2.3. MRI Sequence

Fat infiltration was generally assessed on T1-weighted images, with T2 or fat suppression images often used as adjuncts. Only one quantitative study used the DIXON method to assess fat infiltration [6]. In most cases, edema was evaluated using fat suppression images, mainly STIR, but some studies used T2-weighted images. All quantitative studies were performed using the DIXON method, except one in which the results were not described.

### 2.4. Grading System

As previously mentioned, different grading systems were used in each study. To show how the gradings used in each study are related, a phylogenetic diagram was created according to the reference list of each study (Supplementary Figure S1). In addition, the grading systems included in this systematic review were categorized, and the percentage of each stage in each system was indicated (Supplementary Figure S2).

**Figure 2:**
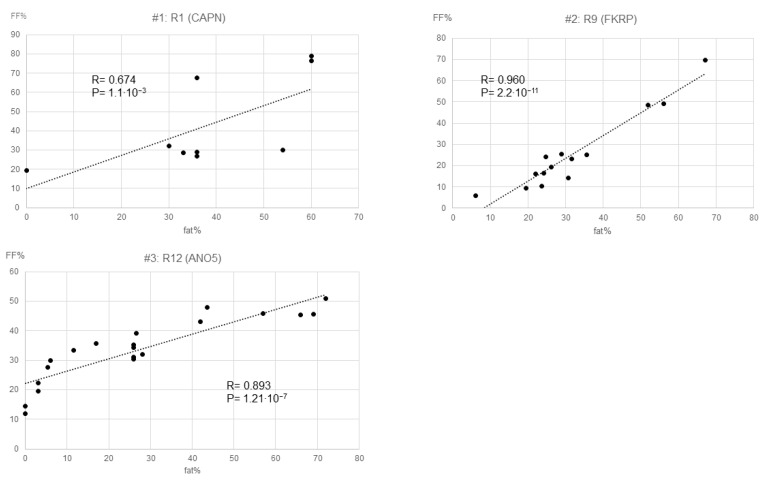
Internal validation of the estimated formula; correlation between FF% and fat%.

Three studies described the results of both grading system and quantitative studies, and all showed a good correlation between FF% and fat% (Figure 2) [6,24,26].

Four of the overall studies used both a grading system and a quantitative study, but one of the studies did not describe the results of the quantitative study. Here we investigated the correlation between the estimated percentage of fat infiltration (fat%) and fat fraction (FF%) for the remaining three studies (#1 for R1(CAPN) [6], #2 for R9(FKRP) [24], #3 for R12(ANO5) [26]). All show very good correlations.

Abbreviations. LGMD: limb-girdle muscular dystrophy, R1 (CAPN): LGMD R1 calpain3-related, R9 (FKRP): LGMD R9 FKRP-related, R12 (ANO5): LGMD R12 anoctamin5-related.

### 2.5. Synthesis and Summary of Results in Each Anatomical Region

**Figure 3:**
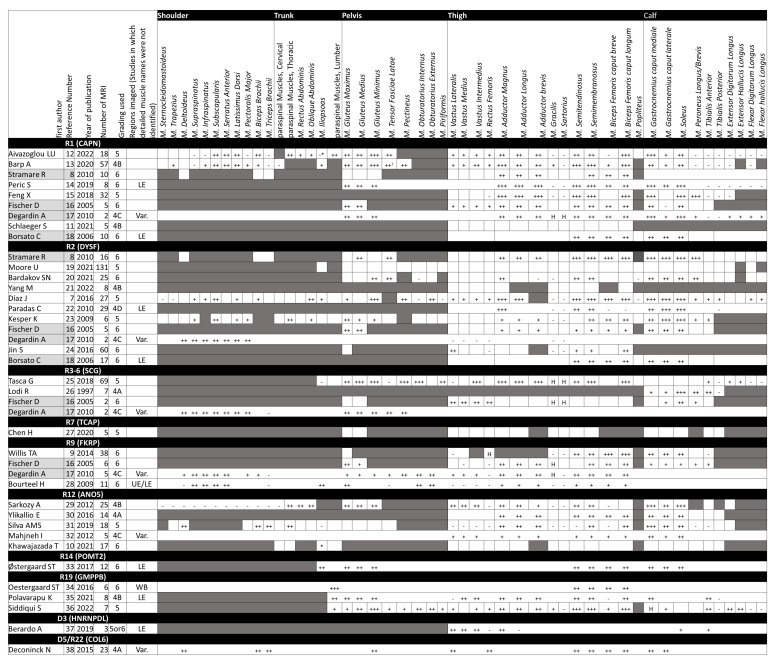
Qualitative synthesis of fatty infiltration.

**Figure 4:**
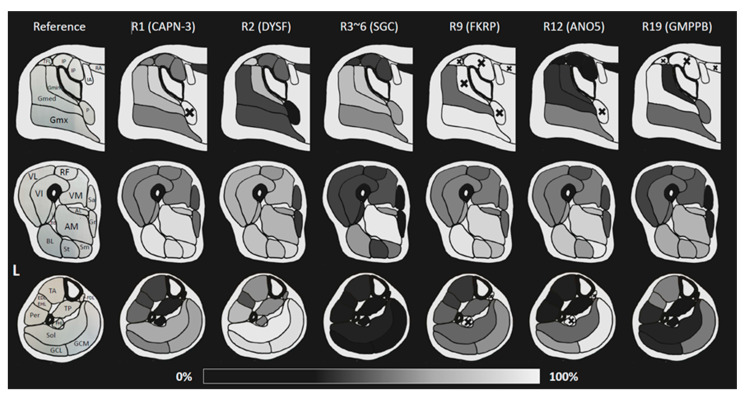
Anatomical heat map resembling MRI axial section image.

The results of the qualitative synthesis are shown in Figure 3, and the quantitative synthesis results in Figure 4. The mean values of fat% and FF% were used to create the heat map, respectively, and the number of samples for each is shown in the Supplementary Table S2. The results for each anatomical site from all the studies are summarized below.

The included studies, the grading system used, the imaging site, and the results are shown for each genotype. If the names of individual muscles are indicated, the corresponding square is surrounded by a thick frame; otherwise, the square is surrounded by a thin frame or no frame. The “Regions imaged” column is used when the imaging area is only roughly known and is left blank when the imaging area is known in detail. If the muscle is clearly not being evaluated, the cells are painted gray. The meaning of each symbol is as follows: Muscles not mentioned by the authors are unmarked, and those considered ‘spared’ (‘not degenerated’) are listed as ‘-’. Muscles with superlative adjectives such as ‘most severely’ and ‘the earliest’ were marked as ‘+++’, the other adjectives such as ‘severely’, ‘early’, etc., were given as ‘++’, and muscles that were only mentioned as having a certain degree of muscle atrophy or fat infiltration was given as ‘+’. When muscle hypertrophy (including pseudohypertrophy) was described, it was listed separately as ‘H’.

* Mentioned as Iliotibialis in the paper.

Gray background indicates multiple genotypes in the same study.

Abbreviations. LGMD: limb-girdle muscular dystrophy, R1 (CAPN): LGMD R1 calpain3-related, R2 (DYSF): LGMD R2 dysferlin-related, R3–6 (SGC): collective name of LGMD R3–6 sarcoglycan-related, R7 (TCAP): LGMD R7 telethonin-related, R9 (FKRP): LGMD R9 FKRP-related, R12 (ANO5): LGMD R12 anoctamin5-related, R14 (POMT2): LGMD R14 POMT2-related, R19 (GMPPB): LGMD R19 GMPPB-related, D3 (HNRNPDL): LGMD D3 HNRNPDL-related, D5/R22 (Col6): combined name of LGMD D5 collagen 6-related and LGMD R22 collagen 6-related, or Bethlem myopathy.

The sFat% (synthesized fat%; the composite of all fat% or FF% in each study) for each muscle in the pelvic, mid-thigh, and mid-lower leg regions are shown in heatmap form. For each genotype, the muscles with the highest fat infiltration were represented in white (100%), the same color as the adipose tissue, and the muscles with the lowest fat infiltration were represented in dark gray, the same color as the bone cortex and background, on a 200-level grayscale. A correspondence figure between the illustration and the muscle name is attached on the left side.

Abbreviations. LGMD: limb-girdle muscular dystrophy, R1 (CAPN): LGMD R1 calpain3-related, R2 (DYSF): LGMD R2 dysferlin-related, R3–6 (SGC): collective name of LGMD R3–6 sarcoglycan-related, R9 (FKRP): LGMD R9 FKRP-related, R12 (ANO5): LGMD R12 anoctamin5-related, R19 (GMPPB): LGMD R19 GMPPB-related, IA: iliac artery, IP: iliopsoas, RA: rectus abdominis, P: piriformis, TFL: tensor fasciae latae, Gmin: gluteus minimus, Gmed: gluteus medius, Gmx: Gluteus maximus, RF: rectus femoris, VL: vastus lateralis, VI: vastus intermedius, VM: vastus medialis, AM: adductor magnus, AL: adductor longus, Sa: sartorius, Gr: gracilis, BB: biceps femoris caput breve, BL: biceps femoris caput longum, St: semitendinosus, Sm: semimembranosus, TA: tibialis anterior, EDL: extensor digitorum longus, EHL: extensor hallucis longus, Per: peroneus (longus/brevis), TP: tibialis posterior, FDL: flexor digitorum longus, FHL: flexor hallucis longus, Sol: soleus, GCL: gastrocnemius caput laterale, GCM: gastrocnemius caput mediale, L: left. The mark ‘╳’ Indicates muscles that have not been sufficiently investigated in any of the included studies.

### 2.6. Pelvis

Atrophy of Gluteus medius (Gmed) muscle causing waddling gait was severe in R3–6 and R19, with relatively mild impairment in R2 and R12. Each genotype characterized the pattern of disability of the Gluteus muscles, although R9 was inaccurate due to insufficient data on the Gluteus minimus (Gmin) muscle. Gmin was most severely affected in R1 and R2, Gmed was most severely affected in R19, and the gluteus maximus (Gmx) was accentuated in R9 and R12. Only Tensor fasciae latae (TFL) was strongly impaired only in R2, which is uncertain due to the small number of studies.

### 2.7. Thigh

The Quadriceps femoris (QF) muscle was basically impaired in any LGMD but preserved in R3–6 in these six disease types. Rectus femoris (RF) tended to be relatively well preserved among the QF muscles, with little intermuscular variation in R1. RF was relatively preserved in R12. The relative preservation of the Vastus lateralis (VL) in R19 was characteristic. Two studies in R1 mentioned pseudocollagen sign in the QF; one study found it in 13/18 [7] and the other in 29/57 [8].

The adductor muscle group was variable in the muscles examined due to the difficulty of differentiation in each image, and intermuscular differences were not evident in any of the diseases. On the other hand, R3–6, where QF was relatively well maintained, had the strongest impairment of this muscle group. The Gracilis (Gr) and Sartorius (Sa) muscles were described as preserved in the six typical types of disease, with pseudohypertrophy sometimes seen in R1, R3–6, and R9 in multiple studies [11,12,21]. Hamstrings (Ham) were strongly impaired in all six types, with few differences. Relatively light impairment was seen in R3–6 if daring to mention it, and the Biceps femoris long head (BFLH) was noticeably impaired in R9.

### 2.8. Calf

In R3–6, the lower leg was rarely involved. In the other genotypes, the Triceps surae (TS) muscle was generally more affected than different compartments of the calf, most notably in R2 and R12. This may be because both genotypes include the Miyoshi type as a phenotype. Intermuscular differences were also noticeable, with a strong involvement of the Soleus (Sol) in R2 and a strong involvement of the gastrocnemius caput mediale (GCM) in R1/R9/R12/R19, most notably in R12. The tibialis anterior (TA), peroneal (Per), and extensor digitorum longus (EDL) muscles were affected in the limited genotypes (R2 and R19). It should be noted, however, that only some studies have examined the Per and EDL.

### 2.9. Upper Limb and Upper Girdle

Although there are few studies reviewed, and both can only be mentioned to a limited extent, degeneration of the Subscapularis (SSc), Latissimus dorsi (LD), and Biceps brachii (BB) muscles, including the Serratus anterior (SA) muscle, is prominent in R1. In contrast, in R2, SA atrophy is less prominent, and degeneration extends to the Supraspinatus (SSP) and Infraspinatus (ISP) muscles. This is consistent with the fact that clinically, a scapula alata is hardly seen in R2 [11]. The other disease types did not yield results worth mentioning.

### 2.10. Body Trunk

In R1, paraspinal discrepancy (later-medial, cauda-cranial gradient) was described in several studies [7,8].

### 2.11. Pattern of Intramuscular Fatty Infiltration

R9 was described as a stripe and reticular, racy fatty infiltration patterns were observed in multiple muscles [9]. It was reported that a central shadow of RF is observed in R12 [29]. Several studies also described left–right asymmetry in R12 [26,27,29].

### 2.12. Myoedema

Only 16 studies assessed edema; even fewer used a grading system, and the assessment methods varied. A synthesis of studies in which edema is mentioned is shown in the Supplementary Figure S3. There were two types of gradings: six- and four-level. The six-level grading was a two-axis grading method based on the anatomical extent of high STIR signal and signal intensity [5,13]. Results were varied, and many studies did not provide details. We could not point out any commonalities beyond that any of the muscles can have a high STIR signal and that the QF, Sa, Gr, TS is prone to edema.

## 3. Method

A systematic review was conducted according to the PRISMA statement [44]. Ethical approval was not required for this systematic review.

### 3.1. Database Search

The following databases were used for the search: MEDLINE and EMBASE. The Cochrane database to check for similar studies. In addition, Opengrey.eu (https://opengrey.eu/, accessed on 24 April 2023) and the Grey Literature Report (https://www.nyam.org/library/collections-and- resources/grey-literature-report/, accessed on 24 April 2023) were used to search the grey literature. The search terms used were “‘Limb-girdle’ AND ‘MRI’” in all databases.

Before starting, we checked PROSPERO (https://www.crd.york.ac.uk/prospero/, accessed on 13 April 2023) for registrations of systematic reviews related to MRI and other imaging studies and muscle diseases and found no ongoing registered protocols.

The following exclusion criteria were used:

(1) Studies not involving human subjects;
(2) Clinical trials;
(3) Studies that excluded clinical information;
(4) Studies not involving conditions classified as LGMD in the ENMC2017 workshop [1];
(5) Studies not dealing with skeletal muscle MRI;
(6) Studies written in languages other than English;
(7) Reviews, Editorials, and Response letters;
(8) Fewer than 3 patients or only 1 family being scanned;
(9) No definitive genetic or histochemical diagnosis.

In addition, this study aims to analyze mainly adult cases. Still, in the case of studies with a mix of adult and pediatric cases, the following criteria were used for inclusion.

(1) More than half of the patients are adults (18 years or older);
(2) If the condition in (1) is unknown or most patients are under 18, the study must be limited to patients at least 10 years of age.

### 3.2. Data Extraction

The following items were extracted. First author, journal name, year of publication, subtype of samples (if applied, described below in detail), genotype of sample(s), legion MRI performed, number of samples in total, number of samples of MRI performed, condition of MRI (tesla, sequences), body area imaged and each number, demographic data (age at examination, sex, disease duration), what is assessed in the image (fat infiltration, myoedema, or quantitative study), assessment method (detailed grading system or quantitative method), description method of results.

The following subtypes were used: LGMD type, Miyoshi Myopathy type (MM), hyper-CKemia (CK), Asymptomatic carrier (ASM), and Proximodistal myopathy (PDM) for LGMD R2 dysferlin-related (R2(DYSF)); R3~R6 for LGMD R3–6 sarcoglycan-related (R3–6 (SGC)); and LGMD, MM, pseudometabolic (PM), and CK in LGMD R12 anoctamin5-related (R12(ANO5)).

Age and disease duration were calculated primarily by extracting the mean and range described by the study authors or by using detailed patient data, if available. When none of the above were available, median values were extracted.

### 3.3. Data Synthesis and Analysis

#### 3.3.1. Fat Infiltration

To evaluate fatty infiltration, we performed two analyses: qualitative and quantitative synthesis. The first was to summarize the qualitative evaluation. This synthetic method is also used in existing systematic reviews and can be compared to these [3,4]. In addition, this synthesis is also necessary because several studies use the grading system as a methodology, but only the authors’ subjective evaluations are included when describing the results. The second, or quantitative synthesis, is to synthesize the objective data—the fat infiltration percentage. The approximate value of fat infiltration percentage (fat%) was calculated from each grading when the grading system evaluated fat infiltration. As mentioned previously [3,4,45], the grading systems used in each study use different definitions and grades, making simple comparisons impossible. To put this into a comparable form, we reconverted the visually defined grading into the estimated percentage of fat infiltration (fat%). For this purpose, we used a numeric scale to percentage conversion formula. Details on these two evaluation methods are described in the Supplementary Material. The internal validity of the approximate formula was verified in papers in which the grading system and quantitative study were described [6,24,26].

We synthesized the means for fat fraction (FF%), for the quantitative studies this is the most common indicator used. In addition, since FF% is considered to be evaluating essentially the same phenomenon as fat%, we also synthesized the means for these two. If both their grading system and quantitative study had been conducted in the same study [6,24,26], the fat% estimated from the grading system was used for the calculation.

Of the final synthesized FF% and fat% averages (synthesized fat%, sFat%), muscles with sufficient sample numbers and included in representative MRI cross-sections (pelvic, mid-thigh, and mid-lower leg) were selected to produce an anatomical heat map. The colors were represented as a grayscale of 200 tones, with the most affected muscle of each genotype in light gray and the density of the bone cortex in black, in a manner that approximates an MRI axial section image.

#### 3.3.2. Myoedema

A descriptive summary of the studies, evaluation methods, and results described for muscle edema.

#### 3.3.3. Additional Analytic Points

In addition to the analyses listed above, the authors of the respective studies may mention other specific points, such as atrophic distribution within the muscle and left—right differences. These points are difficult to synthesize because they are usually not computable and are mentioned in only a single study. However, since this point may contain clinically important points, they are mentioned together for each genotype.

## 4. Discussion

In the present study, we synthesized previous studies on limb-girdle muscular dystrophies in adulthood. We characterized the distribution of affected muscles as expected for the six major forms of the LGMD disease spectrum.

What differentiates this study from previous attempts is that the MRI data itself was integrated rather than the authors’ interpretation of each study by replacing the grading system with fat% in a simulated manner. In other words, there have been two systematic reviews of limb-girdle muscular dystrophies [3,4], both of which differ essentially from the present study in that they are only qualitative syntheses of the interpretations by the authors of each study. The 2017 study was limited in its description because it targeted various muscle diseases, not only LGMDs, and there were not many studies reporting LGMDs MRI patterns at that time [3]. The 2023 study included LGMDs, but included all ages and multiple case reports [4]. Therefore, this study includes several articles that have not been subjected to either quantitative or semi-quantitative analysis, and the authors focus on whether the authors’ stated findings “agree” with their synthesis of the study. One of the strengths of the 2023 study is that it included rare genotypes that could not be addressed in our current study due to limited data.

Based on these differences, the review studies and our present study are considered complementary to each other. However, our present study confirms some former findings, but reports several new additions.

(1). Newly noted in our study, which has not been reported in previous MRI studies:
  #1. Gmed is more likely to be affected in R19 and Gmx in R12;
  #2. TFL is impaired in R2;
  #3. RF is relatively preserved in R12;
  #4. VL is preserved in R19;
  #5. Hypertrophy of Gr/Sar is likely to be observed in R1 and R3–6;
  #6. BFLH impairment is prominent in R9;
  #7. In R2 and R12, lower leg involvement is evident;
  #8. The impairment of Sol in R2 and GCM in R1/R9/R12/R19 is notable, especially in R12;
  #9. R2/R19 is also apt to involve the TA/Per/EDL;
  #10. In R1, SA is impaired, and ISP is preserved, but in R2, the pattern is reversed;
  #11. A specific finding may be the later-medial, cauda-cranial gradient of the paraspinal muscles in R1.

Due to the nature of our study, which integrates the patterns of muscle involvement by each genotype with gradations, many findings have not been seen in previous descriptive studies. Still, it is necessary to be careful whether these findings are plausible. As mentioned earlier, the number of samples for R9/R12/R19, in particular, is considered insufficient for integration and may not accurately reflect the population. On the other hand, findings such as #5, #7, #8, and #10 are probably accurate given the clinical findings. The reliability of each of these findings varies in degree, but all will require further follow-up studies.

(2). Comparison with results of the previous 2023 Systematic Reviews
  The previously described patterns, confirmed in our study:
  #1. Muscle selectivity in the involvement of the gluteus maximus muscle group is very useful for differentiation. Gmin is most likely affected by R1/R2, whereas Gmx is most likely affected by R9;
  #2. It is generally expected to have less involvement of the Sa and Gr muscles (with a few exceptions);
  #3. In R3–6, the lower leg is spared even in the later stages. If impaired, TA is typical;
  #4. In R1/R9, TA is preserved even in the late stage;
  #5. The Gastrocnemius caput laterale (GCL) is relatively preserved in R1.

These are all probably true because they contain a relatively large number of genotypes, and the data are obvious.

(3). Findings mentioned in the previous 2023 studies and not pursued in our study:
  #1. If there is no accentuation of degeneration of the gluteus muscle group, R12/R19 if the gluteus muscle group is less degenerative than other parts of the body, otherwise R9 is the differential [4];
  #2. R12 has a patchy rather than homogeneous distribution of fatty infiltration in the thighs [4];
  #3. Biceps femoris short head (BFSH) will likely be spared in R12 [4];
  #4. TA and tibialis posterior (TP) are key muscles of differentiation in the lower leg; TP is impaired in R19 [4];
  #5. R3–6 shows a concentric fatty infiltration pattern (VL, RF, and Vastus medialis (VM) outer areas are spared, while others have degenerated) [2];
  #6. Concentric fatty infiltration is seen in the distal femur, even in R9 [2].

These findings can be categorized mainly as either <1> the present study was not sufficient in sample number (R9/R12/R19) or <2> those that refer not only to the pattern of muscle damage in each region, such as the thigh and lower leg, but also to the gradations of damage within a single muscle, both of which could still be true simply because they could not be pursued due to the design of this study. Further study is required on this point.

### 4.1. For Genotypes Not Addressed in This Study

In their study, Alawneh et al. also described the findings of rare LGMD genotypes such as R7/R8/R10/R11/R21/D1/D2/D3 [4]. These include patterns not included in the six genotypes in the present study, such as more involvement of the anterior thigh, more involvement of the Sa/Gr, and TP involvement. Naturally, these points have yet to be verified in this study. These rare genotypes require a coherent number of reports from concentration zones or international collaborative studies.

### 4.2. Advantages of Our Systematic Review and Future Recommendations

Our study clarified that some frequent genotypes of LGMDs have a distinctive likelihood of muscle impairment patterns that can be, therefore separated from other LDMD genotypes. Although many studies have been conducted, it was challenging to obtain a comprehensive picture of LGMD. This relieves the clinician from having to analyze skeletal muscle MRIs by reconstructing fragmentary facts from the literature in his or her brain and allows the clinician to grasp the gestalt of the MRI image of each genotype at a glance by referring to Figure 4. Furthermore, qualitative synthesis such as that performed here could be applied to skeletal muscle MRI studies of other myopathies, and similar SR could be performed with extended controls to create a “conceptual atlas” of MRI images across muscle diseases.

Using a uniform evaluation method in future skeletal muscle MRI studies is desirable. In the present study, all the studies (grading system and quantitative study) were integrated in a half-assessed manner, which would have been unnecessary if the studies were carried out under a unified grading system. It is crucial to build a consensus on this issue. Therefore, we suggest the following MRI analytic steps: first, even if it is a case report, at least the skeletal muscle MRI evaluation should be described by a grading system. Otherwise, comparing and integrating the given pattern in future summary reports will be puzzling. Second, at least three muscle compartment sites should be evaluated: pelvic, thigh, and lower leg. More importantly, it is desirable to be capable of evaluating all the muscles of the muscle groups shown in Figure 4 of our study, for example, all three types of gluteal muscles. Third, the grading system should preferably be based on the six-step system proposed by Mercuri [46]. Mercuri grading can be evaluated from 0% to 100% and was applied in two of the three studies that examined correlations with FF% in the present study, which showed excellent correlations. Finally, the upper limb girdle and lumbar spine levels, for which there has been insufficient data, should also be incorporated in future studies. Several previous studies have shown some findings in this area, and more data need to be accumulated in the future, which will increase the level of clinically meaningful pattern recognition results.

### 4.3. Limitations

Our systematic review has several limitations in addition to those already mentioned above. First, there is considerable variation in the items presented in each study, and not all samples have been appropriately synthesized. Second, the position of genetic variation and homo/hetero zygosity of individual cases were not considered in the synthesis. Therefore, there may be more significant variability in individual cases. Third, the number of genotypes for which quantitative synthesis was possible in this study was limited, and it remains possible that they share characteristics with other diseases. Finally, many of the studies addressed in this systematic review evaluated the signal as a muscle or muscle group unit in a single plane. Consequently, there is limited data on the pattern of fat infiltration within the muscle or whether there are gradations such as distal or proximal within the muscle.

## 5. Conclusions

We performed a quantitative MRI finding review synthesis and described the fatty infiltration patterns of the six major genotypes of LGMD. Our summary synthesis reveals a distinct distribution pattern of affected muscles by LGMD genotypes, which may be helpful for a quick first-tier differential diagnosis guiding genetic diagnostics.

## Figures and Tables

**Table 1:** Summary of demographic data of each genotype.

	Qualitative Study (with Grading System)						Quantitative Study (Fat Fraction, FF%)		
	Overall			Fat% Calculation					
	N of Study	N of Case	Synthetic Mean	N of Study	N of Case	Synthetic Mean	N of Study	N of Case	Synthetic Mean
R1 (CAPN)	9	159		7	128		3	35	
Mean age	9	159	33.8	7	128	35.1	3	35	40.9
Mean duration	6	60	14.0	4	38	15.9	3	35	18.9
Male%	8	156	47.7	7	128	48.8	3	35	41.6
R2 (DYSF)	11	364		9			2	21	
Mean age	11	364	34.2	9	333	34.4	1	9	49.4
Mean duration	9	321	12.8	7	292	13.2	1	9	25.7
Male%	9	333	51.3	9	333	51.3	2	21	40.2
R3–6 (SCG)	4	81		3	78				
Mean age	4	81	22.8	3	78	22.5			
Mean duration	4	81	13.2	3	78	12.8			
Male%	3	78	50.0	3	78	50.0			
R9 (FKRP)	4	62		2	44		4	70	
Mean age	4	62	38.4	2	44	40.6	3	63	39.4
Mean duration	4	62	20.0	2	44	19.3	3	63	19.5
Male%	3	55	49.1	2	44	43.3	4	70	50.0
R12 (ANO5)	5	98		5	98		2	41	
Mean age	5	98	48.0	5	98	48.0	2	41	47.1
Mean duration	5	98	13.4	5	98	13.4	2	41	15.7
Male%	5	98	71.4	5	98	71.4	2	41	73.1
R19 (GMPPB)	3	25							
Mean age	3	25	42.4						
Mean duration	3	25	20.0						
Male%	3	25	52.0						

Abbreviations. N: number, fat%: estimated percentage of fat infiltration, FF%: fat fraction. R1 (CAPN): LGMD R1 calpain3-related, R2 (DYSF): LGMD R2 dysferlin-related, R3–6 (SGC): collective name of LGMD 3–6 sarcoglycan-related, R9 (FKRP): LGMD R9 FKRP-related, R12 (Ano5): LGMD R12 anoctamin5-related, R19 (GMPPB): LGMD R19 GMPPB-related.

## Data Availability

Data sharing is not applicable to this article because there are no original data. We can share the data set used to analyze, on a proper request.

## Supplementary Materials

The following supporting information can be downloaded at: https://www.mdpi.com/article/10.3390/muscles2040029/s1. Figure S1: grading system history. Figure S2: Relationship between each grade and estimated fat% in each grading system. Figure S3: Qualitative synthesis of myoedema. Table S1: demographic data. Table S2: Quantitative synthesis.
