# Supplementary material for: A Straightforward Approach to Analyze Skeletal Muscle MRI in Limb-Girdle Muscular Dystrophy for Differential Diagnosis: A Systematic Review
Source: Muscles. 2023 Nov 8;2(4):374–88. doi: 10.3390/muscles2040029 (PMC12225514; doi:10.3390/muscles2040029)
Supplement: Supplementary file 1 [file muscles-02-00029-s001.zip › Figure S1_Grading system history.pdf]

Figure S1_grading system history

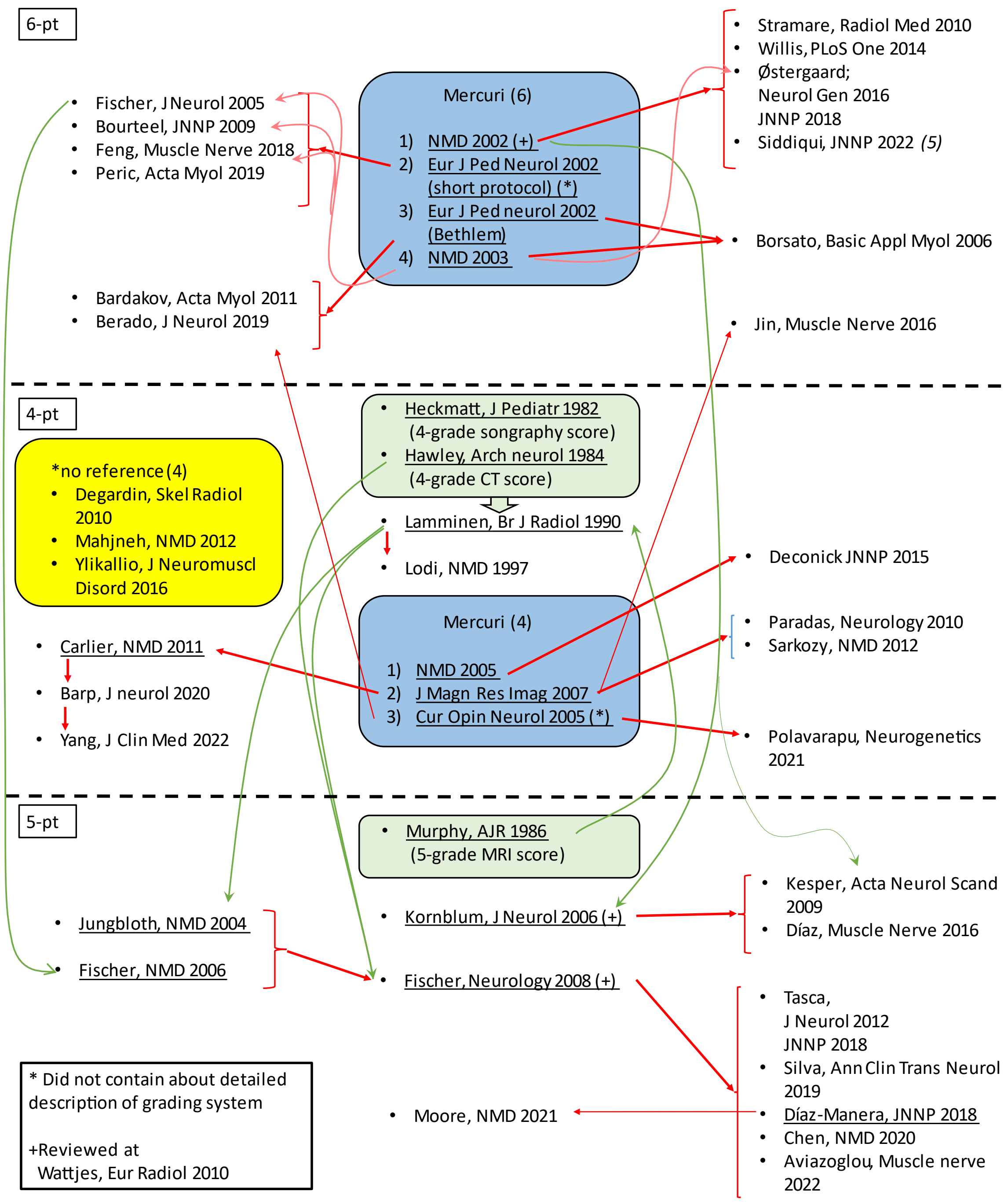

In this figure, the studies are divided into three groups according to the grading system used in each study (4-6 grades), and the relationship is shown by following the references of each study. Those studies that are cited but not related to the actual study are indicated by green arrows, while others are indicated by red arrows. Mercuri's work, which is probably the most widely cited, is shown with a blue background, and the echo, CT, and MRI literature from the 1980s, which may have been the origin of grading, is shown with a light green background. Numbers in italicized parentheses indicate that a different grading is actually used, although they were classified there based on the way they were cited. Studies not included in the present systematic review are underlined.
