# Supplementary material for: A Straightforward Approach to Analyze Skeletal Muscle MRI in Limb-Girdle Muscular Dystrophy for Differential Diagnosis: A Systematic Review
Source: Muscles. 2023 Nov 8;2(4):374–88. doi: 10.3390/muscles2040029 (PMC12225514; doi:10.3390/muscles2040029)
Supplement: Supplementary file 1 [file muscles-02-00029-s001.zip › Figure S2_Each grading system and nomogram.pdf]

Supplementary Figure 2, Relationship between each grade and estimated fat% in each grading system

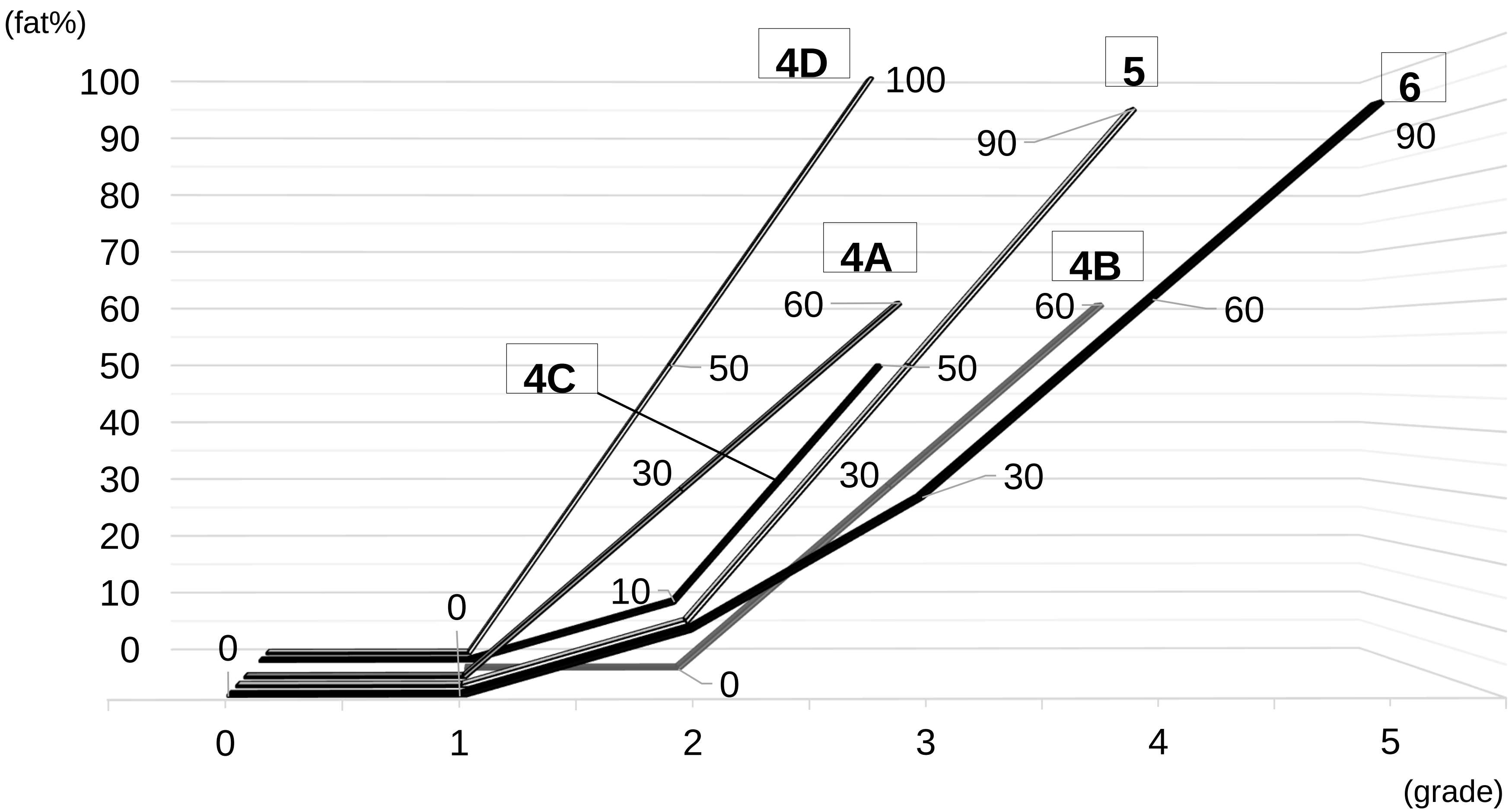

In each grading system, we indicated where each grade value was located in fat%. It is assumed that there is a linear relationship between fat% and each grade. There were six grading systems in total, and especially when a four-step grading system was used, there were four different patterns of significance. The grading system of each study was determined by referring to the expressions in the text and the references.
