# Supplementary material for: A Straightforward Approach to Analyze Skeletal Muscle MRI in Limb-Girdle Muscular Dystrophy for Differential Diagnosis: A Systematic Review
Source: Muscles. 2023 Nov 8;2(4):374–88. doi: 10.3390/muscles2040029 (PMC12225514; doi:10.3390/muscles2040029)
Supplement: Supplementary file 1 [file muscles-02-00029-s001.zip › Figure S3_edema qualitative synthesis.pdf]

Figure S2, Qualitative synthesis of myoedema

For each genotype, the included studies, the grading system used, the imaging site, and the results are shown. If the name of the individual muscle included in the image is known, the corresponding square is surrounded by a thick frame; otherwise, the square is surrounded by a thin frame or no frame. If the muscle is clearly not being evaluated, the cells are painted gray. The meaning of each symbol is as follows; Muscles not mentioned by the authors are unmarked, and those considered 'spared' are listed as '-'.' Muscles with the adjectives such as 'severely', 'early', etc., were given as '++', and muscles that were only mentioned as having certain degree of myoedema were given as '+'. Gray background indicates multiple genotypes in the same study.

							Shoulder									Trunk						Pelvis								Thigh														Calf									
first author	Reference Number	Year of publication	Number of MRI	Sequence	Grading used	detailed presentation	M. Deltoideus	M. Supraspinatus	M. Infraspinatus	M. Subscapularis	M. Serratus Anterior	M. Latissimus Dorsi	M. Pectoralis Major	M. Biceps Brachii	M. Triceps Brachii	paraspinal Muscles, Cervical	paraspinal Muscles, Thoracic	M. Rectus Abdominis	M. Oblique Abdominis	M. Iliopsoas	paraspinal Muscles, Lumber	M. Gluteus Maximus	M. Gluteus Medius	M. Gluteus Minimus	M. Tensor Fasciae Latae	M. Pectineus	M. Obturatorius Internus	M. Obturatorius Externus	M. Piriformis	M. Vastus Lateralis	M. Vastus Medius	M. Vastus Intermedius	M. Rectus Femoris	M. Adductor Magnus	M. Adductor Longus	M. Adductor brevis	M. Gracilis	M. Sartorius	M. Semitendinosus	M. Semimembranosus	M. Biceps Femoris caput breve	M. Biceps Femoris caput longum	M. Popliteus	M. Gastrocnemius caput mediale	M. Gastrocnemius caput laterale	M. Soleus	M. Peroneus Longus/Brevis	M. Tibialis Anterior	M. Tibialis Posterior	M. Extensor Digitorum Longus	M. Extensor Hallucis Longus	M. Flexor Digitorum Longus	M. Flexor hallucis Longus
**R1 (CAPN)**																																																					
Aivazoglou LU	12	2022	18	STIR	4	text																								+	+	+	+											+	+			+	+				
Stramare R	8	2010	10	STIR	6	graph																												-	-	-			++	++	++	++											
Degardin A	17	2010	2	STIR	N/A	none																																															
Borsato C	18	2006	10	STIR	6	graph																								++	++	++	++															+					
**R2 (DYSF)**																																																					
Stramare R	8	2010	16	STIR	6	graph									++															++	++	++	++																				
Bardakov SN	20	2021	25	STIR	N/A	text																+	+	-			+	+		++	++	++	++					++	-	-		++	++	-	-			++	++	++		++	
Yang M	21	2022	8	ND	4	graph																																															
Díaz J	7	2016	27	STIR	N/A	text		++	++			+	+	+	+					++	+	+		-		++		++		++	+	+	++	++	++		++	++	+	+	+	+	+			++	+	+	+			+	+
Paradas C	22	2010	29	STIR	N/A	text																												++										++									
Kesper K	23	2009	6	SPAIR	N/A	text		+		++		+	+					++	+		++	++												+						+	+	+					+	+					
Degardin A	17	2010	2	STIR	N/A	text	"diffuse"																																														
Jin S	24	2016	60	SPAIR	5	graph																								+	++	+		+			-	-															
Borsato C	18	2006	18	STIR	6	graph																																															
**R4 (SCG)**																																																					
Degardin A	17	2010	2	STIR	N/A	text									++																																						
**R9 (FKRP)**																																																					
Degardin A	17	2010	5	STIR	N/A	text																																															
**R12 (ANO5)**																																																					
Sarkozy A	29	2012	25	STIR	N/A	text																									++							++						++		++							
Ylikallio E	30	2016	14	STIR	4	graph																								++	++	++	++	++	++	++			++	++	++	++		++	++	++							
Silva AMS	31	2019	18	STIR	N/A	text																									++							++						++	++	++							
Mahjneh I	32	2012	5	STIR	4	graph								+											+					+	+	+	+	++	++	++			+	++	+	+		++	++	++							
**R19 (GMPPB)**																																																					
Polavarapu K	35	2021	8	STIR	6	text																																										++					
Siddiqui S	36	2022	7	STIR	6	text																								++	++				++		++							++				++	++				++

Abbreviations. LGMD: limb-girdle muscular dystrophy, R1 (CAPN): LGMD R1 calpain3-related, R2 (DYSF): LGMD R2 dysferlin-related, R4 (SGC): LGMD R4 β-sarcoglycan-related, R9 (FKRP): LGMD R9 FKRP-related, R12 (Ano5): LGMD R12 anoctamin5-related, R19 (GMPPB): LGMD R19 GMPPB-related, N/A: not applicable
