# Supplementary material for: A Straightforward Approach to Analyze Skeletal Muscle MRI in Limb-Girdle Muscular Dystrophy for Differential Diagnosis: A Systematic Review
Source: Muscles. 2023 Nov 8;2(4):374–88. doi: 10.3390/muscles2040029 (PMC12225514; doi:10.3390/muscles2040029)
Supplement: Supplementary file 1 [file muscles-02-00029-s001.zip › Muscles_Supple_Method.pdf]

Supplementary material: Qualitative and Quantitative synthesis methods

Qualitative synthesis

To be as objective as possible, a structured qualitative evaluation was conducted in the synthesis. A six-grade scale was used here. Muscles not mentioned by the authors are unmarked, and those considered 'spared' ('not degenerated') are listed as '-'.' Muscles with superlative adjectives such as 'most severely' and 'the earliest' were marked as '+++', the other adjectives such as 'severely', 'early', etc., were given as '++', and muscles that were only mentioned as having a certain degree of muscle atrophy or fat infiltration was given as '+'. When muscle hypertrophy (including pseudohypertrophy) was described, it was listed separately as 'H'. When a muscle group was described rather than a specific muscle, all muscles included in the group were given the same rating.

Quantitative synthesis

1) If a study's method or relevant reference describes the relationship between each grading and %, it is given priority.
2) If 1) is not stated, set as follows; 'Normal'= 0%, 'mildly'=10%, 'moderately'=30%, 'severely' or 'moderate to severe'=60%, 'washed-out' or 'confluent'=90%, 'complete' or 'end-stage'=100%.
3) Basically, the grading number itself is used as is for statistical processing, but when grading such as 2a/2b is used, points are assigned as follows; grade0=0, grade1=1, grade2a=2, grade2b=3, grade3=4, grade4=5 [7, 8].
4) Convert from the score given for each grading to a percentage according to the definitions in 1)-3). The fat infiltration was assumed to increase linearly from each grade to the next.

If the grading was given for each sample, the original number was used, and if only the mean value and the number of samples were given, the product of the two was used to calculate the overall mean value. Studies with only qualitative evaluation were excluded.
