# Supplementary material for: A Straightforward Approach to Analyze Skeletal Muscle MRI in Limb-Girdle Muscular Dystrophy for Differential Diagnosis: A Systematic Review
Source: Muscles. 2023 Nov 8;2(4):374–88. doi: 10.3390/muscles2040029 (PMC12225514; doi:10.3390/muscles2040029)
Supplement: Supplementary file 1 [file muscles-02-00029-s001.zip › Table S1_demographic data.pdf]

Supplementary Table 1. demographic data and summary of study method

**Genotype**					N		MRI condition			Age			Sex		Duration			Assessment			Fat		Edema		Quantitative	
First author	Y	Ref	Phenotype	Region	T	MRI	Tesla	Sequences	Lesion	Range	Mean	SD	M	M %	Range	Mean	SD	FG	E	Q	G	Result	G	Result	Method	Result
**R1 (CAPN)**																										
Aivazoglou LU	2022		LGMD	BRA	18	18	1.5	T1, STIR	WB	12~68	35.4	16.0	9	50.0	1~34	16.3	9.7	+	+		5	heatmap	4	NID		
Barp A	2020		LGMD	EUR	57	57	1,5-3	T1	LE(52), other(28)	7~78	38.4	15.8	20	35.0	N/D	N/D	N/D	+			4B	heatmap				
Stramare R	2010		LGMD	ITA	10	10	1.5	T1, TIRM	WB	17~65	32.0	N/D	8	80.0	N/D	N/D	N/D	+	+		6	graph	6	graph		
Peric S	2019		LGMD	SRB	19	8	N/D	T1, T2, PD, DIXON	LE	9~37	25.4	10.4	8	42.0	0~19	9.8	5.9	+			6	NID				
Feng X	2018		LGMD	CHN	32	32	3	T1	UL(28), C(29)	12~52	27.0	9.6	18	56.3	N/D	N/D	N/D	+			5	graph				
Fischer D	2005		LGMD	EUR	5	5	1.5	T1, SPIR	LE	18~57	35.6	15.9	2	40.0	8~20	16.0	6.7	+	+		6	table		NID		
Degardin A	2010		LGMD	FRA	3	2	N/D	T1, STIR	Various	N/D	36.7	7.4	N/D	N/D	N/D	16.0	2.0	+	+		4C	NID	—	NID		
Schlaeger S	2021		LGMD	DEU	5	5	3	DIXON	UL	26~84	55.6	14.7	4	30.8	1~32	9.5	9.5	+		+	4B	table			FF	heatmap
Borsato C	2006		LGMD	ITA	10	10	1	T1, T2, STIR	LE	17-60	32.0	N/D	8	80.0	N/D	18.3	N/D	+	+		6	graph	6	graph		
Arrigoni F	2018		LGMD	ITA	11	11	3	T1, T2, DIXON, SPIR	UL	22~51	36.5	9.8	4	36.4	13~30	20.6	6.7			+					FF, T2fat, FA, MD	graph
Forsting J	2022		LGMD	DEU	19	19	3	DIXON, MESE, DW, SPIR	LE	25~68	39.5	13.3	9	47.4	10~42	20.3	7.4			+					FF, FA, MD, RD, SNR	heatmap
**R2 (DYSF)**																										
Stramare R	2010		LGMD	ITA	16	16	1.5	T1, TIRM	WB	16~50	33.0	N/D	13	81.3	N/D	N/D	N/D	+	+		6	graph	6	graph		
Moore U	2021		LGMD(114), MM(54)	Jain F	168	131	N/D	T1	LE(59), C(72)	11~86	36.7	N/D	75	44.6	1-51	15.9	N/D	+			5	graph				
Bardakov SN	2021		LGMD(14), MM(11)	RUS	25	25	1.5	T1, T2, STIR, T2MSME	LE	N/D	37.0	N/D	16	64.0	N/D	N/D	N/D	+	+	+	6	graph	—	graph	FF, T2t	N/D
Yang M	2022		LGMD	CHN	8	8	N/D	T1, T2 (N/D in method)	Th	24~59	32.0	N/D	5	62.5	0~5	1.5	N/D	+	+		4B	graph	4	graph		
Díaz J	2016		LGMD(7)/MM(20)	CHL	27	27	1.5	T1, STIR	WB	19~51	30.9	7.6	12	44.4	2~24	10.7	5.6	+	+		5	graph	—	NID		
Paradas C	2010		LGMD(12), MM(14), ASC(3)	ESP	29	29	1.5	T1, STIR	LE	2~72	32.3	16.8	N/D	N/D	0~34	8.3	9.8	+	+		4D	NID	—	NID		
Kesper K	2009		LGMD(2), MM(3), CK(1)	DEU	6	6	3	T1, T2, SPAIR	WB	26~48	35.0	8.0	4	66.7	9~18	11.3	6.6	+	+		5	table	—	NID		
Fischer D	2005		LGMD	EUR	5	5	1.5	T1, SPIR	LE	27~39	32.2	4.8	2	40.0	8~21	13.4	5.0	+	+		6	table	—	NID		
Degardin A	2010		LGMD	FRA	2	2	N/D	T1, STIR	Various	N/D	35.5	13.4	N/D	N/D	N/D	13.5	4.9	+	+		4C	NID	—	NID		
Jin S	2016		LGMD(31), MM(23), CK(2), PDM(4)	CHN	60	31	3	T1, SPAIR	T	16~51	29.4	7.4	30	50	0.17-25	8.2	5.7	+	+		6	graph	5	graph		
Borsato C	2006		LGMD	ITA	18	17	1	T1, T2, STIR	LE	16-50	33.0	N/D	14	77.8	N/D	14.8	N/D	+	+		6	graph	6	graph		
Arrigoni F	2018		LGMD	ITA	9	9	3	T1, T2, DIXON, SPIR	T	34~57	49.4	7.3	5	55.6	12~31	25.7	5.8			+					FF, T2fat, FA, MD	graph
Reyngoudt H	2021		LGMD/MM (not detailed)	FRA	12	12	3	DIXON	LE	N/D	36.7*	N/D	6	50.0	N/D	19.1*	N/D			+					FF	table
**R3-6 (SGC)**																										
Tasca G	2018		LGMD- R3(38), R4(12), R5(18), R6(1)	EUR	69	69	1.5	T1	LE	4~59	22.8	15.7	34	49.3	0~47	12.9	11.3	+			5	heatmap				
Lodi R	1997		LGMD	GBR	7	7	2	T1, T2	LE	11~25	15.3	4.7	4	57.1	3~12	7.3	3.0	+			4A	table				
Fischer D	2005		LGMD-R3	EUR	2	2	1.5	T1, SPIR	LE	12~60	36.0	33.9	1	50.0	10~46	28.0	25.5	+	+		6	table	—	NID		
Degardin A	2010		LGMD- R4(1), R5(2)	FRA	3	1	N/D	T1, STIR	Various	N/D	30.3	2.1	N/D	N/D	N/D	24.0	4.0	+	+		4C	NID	—	NID		
**R7 (TCAP)**																										
Chen H	2020		LGMD	CHN	5	5	3	T1, T2, STIR	LE	28~47	34.0	7.5	3	60.0	11~30	18.0	7.2	+			5	table				
**R9 (FKRP)**																										
Willis TA	2014		LGMD	EUR	38	38	3	T1, DIXON	T	18~64	40.7	12.5	19	50.0	0~49	18.6	10.1	+		+	6	table			FF	table
Fischer D	2005		LGMD	EUR	6	6	1.5	T1, SPIR	LE	32~48	40.2	6.1	3	50.0	0-35	23.5	12.2	+	+		6	table	—	NID		
Degardin A	2010		LGMD	FRA	7	5	N/D	T1, STIR	Various	N/D	34.6	13.8	N/D	N/D	N/D	19.7	12.8	+	+		4C	NID	—	NID		
Bourteel H	2009		LGMD	FRA	11	11	1.5	T1, T2	UE-LE	N/D	32.0	15.0	5	45.5	2.5~45	23.0	11.0	+			6	NID				
Revsbech KL	2022		LGMD	DNK	14	14	3	T1, DIXON	WB	19~60	36.4	11.8	6	42.9	6~46	21.5	11.8			+					FF	graph
Løkken N	2016		LGMD	DNK	11	11	3	T1, DIXON	LL	22~62	38.9	12.8	8	72.7	5~41	20.3	9.3			+					FF, CSA, CCSA	graph
Reyngoudt H	2021		LGMD	FRA	7	7	3	DIXON	LE	N/D	40.9*	N/D	2	28.6	N/D	26.9*	N/D			+					FF	table
**R12 (ANO5)**																										
Sarkozy A	2012		LGMD(12), PM(3), MM(4), CK(6)	EUR	25	25	N/D	T1, STIR	LE	11~69	44.2	15.2	20	80.0	0~29	12.0	8.9	+	+		4B	table	—	NID		
Ylikallio E	2016		LGMD(5), PM(7), MM(1), CK(1)	FIN	14	14	1.5	T1, STIR	LE	31~69	54.4	9.9	8	57.1	6~48	22.3	12.8	+	+		4A	table	4	table		
Silva AMS	2019		LGMD(25), PM(7), CK(4), MM(1)	BRA	37	18	N/D	T1, STIR	WB(7), LE(11)	12~72	45.8	16.5	27	73.0	0~20	8.9	5.5	+	+		5	heatmap	—	NID		
Mahjneh I	2012		PM(1), MM(4)	FIN/NLD	5	5	<1.5	T1, T2, STIR	various	29~73	51.8	19.5	5	100	9~23	15.6	6.6	+	+		4C	table	4	table		
Khawajazada T	2021		LGMD(13), PM(2), CK(2)	DNK	17	17	3	T1, DIXON	WB	31~73	51.8	10.4	10	58.8	1~42	17.4	16.3	+		+	6	graph			FF	Graph
De Wel B	2022		LGMD/MM(15), PM(9)	BEL	24	24	1.5	T1, DIXON	WB	19~70	43.8	13.9	20	83.3	2~33	14.5	10.8			+					FF	table
**R14 (POMT2)**																										
Østergaard ST	2017		LGMD	EUR	12	12	N/D	T1	LE	17~60	33.2	15.9	5	41.7	3~54	24.3	16.1	+			6	NID				
**R19 (GMPPB)**																										
Oestergaard ST	2016		LGMD	DNK/FRA	6	4	N/D	T1	WB	27~51	37.5	11.9	3	50.0	10~36	18.8	11.7	+			6	graph				
Polavarapu K	2021		LGMD	IND	12	8	1.5	T1, T2, STIR	LE	27~54	40.7	8.2	6	50.0	2~39	17.6	10.7	+	+		4B	NID	6	NID		
Siddiqui S	2022		LGMD	IND	7	7	1.5	T1, T2, STIR	LE	32~63	49.6	10.8	4	57.1	10~41	25.0	11.5	+	+		5	graph	6	NID		
**D3 (HNRNPDL)**																										
Berardo A	2019		LGMD	ARG	5	3	N/D	T1, STIR	LE	56~68	58.8	8.6	3	60.0	2~21	13.2	9.0	+	+		5or6	NID	—	NID		
**D5/R12 (COL6)**																										
Deconinck N	2015		Bethlem	FRA	35	23	N/D	T1	WB(7), LE(16)	14~75	40.2	17.7	14	40.0	4~69	30.5	18.4	+			4A	NID				
Salim R	2020		Bethlem	DNK	15	15	3	T1, DIXON	WB(5), LE(10)	13~57	34.6	13.9	11	73.3	10~54	26.3	14.9			+					FF	graph

* The value is median value, not mean
Gray background indicates multiple genotypes in the same study.

Abbreviations.
LGMD: limb-girdle muscular dystrophy, R1 (CAPN): LGMD R1 calpain3-related, R2 (DYSF): LGMD R2 dysferlin-related, R3-6 (SGC): collective name of LGMD R3-6 sarcoglycan-related, R7 (TCAP): LGMD R7 telethonin-related, R9 (FKRP): LGMD R9 FKRP-related, R12 (Ano5): LGMD R12 anoctamin5-related, R14 (POMT2): LGMD R14 POMT2-related, R19 (GMPPB): LGMD R19 GMPPB-related, D3 (HNRNPDL): LGMD D3 HNRNPDL-related, D5/R22 (Col6): combined name of LGMD D5 collagen 6-related and LGMD R22 collagen 6-related, or Bethlem myopathy, Y: year of publication, MM: Myopathy type, CK: hyper-CKemia, ASM: Asymptomatic career, PDM: Proximodistal myopathy (for dysferlinopathy), PM: pseudometabolic subtype (for anoctamin-5 mutation), ARG: Argentina, BEL: Belgium, BRA: Brazil, CHL: Chile, CHN: China, DEU: Germany, DNK: Denmark, ESP: Spain, EUR: multicenter study in European continent, FIN: Finland, FRA: France, GBR: United kingdom, IND: India, ITA: Italy, Jain F: International study of Jain foundation, NLD: Netherland, RUS: Russia, SRB: Serbia, N: number, T: total, N/D: not described, WB: whole body, LE: lower extremities, UL: upper and lower extremities, C: calf, T: thigh, UE: upper extremities, M: male, FG: grading of fat infiltration, E: Myoedema, Q: quantitative assessment: G: grading system, NID: not in detail, FF: fat fraction, FA: fractional anisotropy, MD: mean diffusivity, RD: radial diffusivity, SNR: signal to noise ratio, CSA: cross-sectional area, CCSA: contractile CSA.
