# Supplementary material for: A Straightforward Approach to Analyze Skeletal Muscle MRI in Limb-Girdle Muscular Dystrophy for Differential Diagnosis: A Systematic Review
Source: Muscles. 2023 Nov 8;2(4):374–88. doi: 10.3390/muscles2040029 (PMC12225514; doi:10.3390/muscles2040029)
Supplement: Supplementary file 1 [file muscles-02-00029-s001.zip › Table S2_Quantitative synthesis.pdf]

Supplementary Table. The quantitative synthesis; mean values of fat%, FF%, sFat% and each numbers

	R1						R2						R3-6		R9						R12						R19	
muscle	n(fat%)	mean fat%	n(FF%)	mean FF%	total n	sFat%	n(fat%)	mean fat%	n(FF%)	mean FF%	total n	sFat%	n(fat%)	mean fat%	n(fat%)	mean fat%	n(FF%)	mean FF%	total n	sFat%	n(fat%)	mean fat%	n(FF%)	mean FF%	total n	sFat%	n(fat%)	mean fat%
Rectus abdominis	18	7.2			18	7.20	33	8.18			33	8.18	60	1							18	0			18	0.00		
Iliopsoas	62	25.47			62	25.47	49	19.55			49	19.55	62	10							23	0			23	0.00		
Lumbar erector spinae	18	56.8			18	56.80	27	50			27	50.00	52	30							35	11.03			35	11.03	13	63.43
Gluteus Maximus	85	26.35			85	26.35	169	12.95			169	12.95	64	32.33	44	64			44	64.00	52	3.85	24	38.1	76	14.67	7	32.8
Gluteus Medius	85	42.14			85	42.14	138	11.62			138	11.62	64	47.91	6	23.33			6	23.33	47	3.46			47	3.46	7	90
Gluteus Minimus	80	50.72			80	50.72	133	45.22			133	45.22	62	51.2							52	4.16			52	4.16	7	6.4
Tensor Fascia Latae	70	27.66			70	27.66	90	51.46			90	51.46	65	7.2							23	0			23	0.00		
Piriformis							92	0			92	0.00	62	38													7	32.8
Vastus lateralis	128	22.53	30	46.55	158	27.09	194	42.15	21	47.66	215	42.69	71	10.99	44	29.69	21	29.9	65	29.76	83	10.41	24	28.5	107	14.47	13	16.83
Vastus medialis	128	22.89	30	45.32	158	27.15	194	43.91	21	49.2	215	44.43	71	15.3	44	27.71	21	31.57	65	28.96	83	9.52	24	32.6	107	14.70	13	24.58
Vastus intermedius	128	27.29	11	52	139	29.25	194	42.05	21	50.4	215	42.87	71	10.99	6	23.33	21	33.6	27	31.32	83	8.85	24	29.9	107	13.57	13	33.85
Rectus femoris	123	21.66	30	42.88	153	25.82	189	34.12	21	43.6	210	35.07	69	8	38	19.48	21	23.3	59	20.84	83	3.43	24	21.6	107	7.51	13	30.83
Adductor magnus	113	52.69	11	61.5	124	53.47	177	43.41	21	53.61	198	44.49	71	61.17	6	55	21	59.13	27	58.21	83	20.62	24	49.3	107	27.05	7	67.2
Adductor Longus	80	43.48	11	61.5	91	45.66	164	34.73	21	51.56	185	36.64	67	35.6	0		7	29.8	7	29.80	83	19.47	24	37.6	107	23.54	7	67.2
Gracilis	108	12.37	30	52.16	138	21.02	156	3.61	21	30.89	177	6.85	71	0	44	20.93	21	23.33	65	21.71	83	6.02	24	13.8	107	7.77	7	3.6
Sartorius	103	4.92	30	45.23	133	14.01	148	7.71	21	40.5	169	11.78	69	0	38	24.78	21	28.27	59	26.02	83	2.29	24	17.6	107	5.72	7	0
Semitendinosus	123	48.1	30	68.66	153	52.13	189	46.29	21	49.73	210	46.63	69	10	38	56.13	21	49.83	59	53.89	83	14.07	24	33.7	107	18.47	13	59.29
Semimembranosus	128	50.25	30	67.13	158	53.46	194	53.81	21	54.3	215	53.86	71	26.11	44	51.72	21	39.4	65	47.74	83	21.45	24	61.7	107	30.48	13	62.31
Biceps femoris short head	128	24.81	30	61.12	158	31.70	155	38.31	21	52.87	176	40.05	71	5.32	44	33.11	21	54.5	65	40.02	83	15.98	24	27.1	107	18.47	13	31.51
Biceps femoris long head	128	53.32	30	61.12	158	54.80	194	47.78	21	52.87	215	48.28	71	36.61	44	66.19	21	54.5	65	62.41	66	13.98	24	56.6	90	25.35	13	62.31
Medial gastrocnemius	123	34.39	19	58.03	142	37.55	227	56.65	12	48.4	239	56.24	78	0	44	33.48	32	39.65	76	36.08	83	30.12			83	30.12	13	40.68
Lateral gastrocnemius	123	25.33	19	46.4	142	28.15	227	55.65	12	34.8	239	54.60	77	0	44	25.42	32	32.48	76	28.39	83	18.8			83	18.80	7	6.4
Soleus	123	36.5	19	56.97	142	39.24	227	60.47	12	43.3	239	59.61	78	2.82	44	22.24	32	23.59	76	22.81	83	11			83	11.00	7	5
Peroneus Longus	80	15.77	19	41.71	99	20.75	194	45.87	12	44.3	206	45.78	78	1.08	44	19.03	32	24.85	76	21.48	54	0.56			54	0.56	7	7.1
Tibialis anterior	123	9.42	19	17.2	142	10.46	227	33.65	12	26.5	239	33.29	78	3.58	44	5.23	32	12.98	76	8.49	78	1.09			78	1.09	13	42.66
Tibialis posterior	98	1.01	19	11.4	117	2.70	183	32.07	12	29.5	195	31.91	76	0			32	21.3	32	21.30	49	0			49	0.00	7	0
Extensor digitorum longus	46	2.39	19	29.87	65	10.42	183	12.71	12	51.3	195	15.08	69	0.9			7	10	7	10.00	18	0			18	0.00	7	44.4
Flexor Difitorum Longus	18	0			18	0.00	183	27.17			183	27.17	69	0													7	0

Abbreviations: R1: LGMD R1 calpain3-related, R2: LGMD R2 dysferlin-related, R3-6: collective name of LGMD R3-6 sarcoglycan-related,
R9: LGMD R9 FKRP-related, R12 (Ano5): LGMD R12 anoctamin5-related, R19: LGMD R19 GMPPB-related,
fat%: percentage of fat infiltration estimated by grading assessments, FF%: fat fraction,
sFat%: overall percentage of fat infiltration by synthesizing FF% and fat% averages
